# Modulating the RNA Processing and Decay by the Exosome: Altering Rrp44/Dis3 Activity and End-Product

**DOI:** 10.1371/journal.pone.0076504

**Published:** 2013-11-12

**Authors:** Filipa P. Reis, Ana Barbas, A. A. Klauer-King, Borislava Tsanova, Daneen Schaeffer, Eduardo López-Viñas, Paulino Gómez-Puertas, Ambro van Hoof, Cecília M. Arraiano

**Affiliations:** 1 Instituto de Tecnologia Química e Biológica - ITQB, Universidade Nova de Lisboa, Oeiras, Portugal; 2 Department of Microbiology and Molecular Genetics, and The University of Texas Graduate School of Biomedical Sciences, University of Texas Health Science Center-Houston, Houston, Texas, United States of America; 3 Centro de Biologia Molecular “Severo Ochoa” (CSIC-UAM), Campus Universidad Autonoma de Madrid, Madrid, Spain; 4 Biomol-Informatics SL, Madrid, Spain; National Institute for Medical Research, Medical Research Council, London, United Kingdom

## Abstract

In eukaryotes, the exosome plays a central role in RNA maturation, turnover, and quality control. In *Saccharomyces cerevisiae*, the core exosome is composed of nine catalytically inactive subunits constituting a ring structure and the active nuclease Rrp44, also known as Dis3. Rrp44 is a member of the ribonuclease II superfamily of exoribonucleases which include RNase R, Dis3L1 and Dis3L2. In this work we have functionally characterized three residues located in the highly conserved RNB catalytic domain of Rrp44: Y595, Q892 and G895. To address their precise role in Rrp44 activity, we have constructed Rrp44 mutants and compared their activity to the wild-type Rrp44. When we mutated residue Q892 and tested its activity *in vitro*, the enzyme became slightly more active. We also showed that when we mutated Y595, the final degradation product of Rrp44 changed from 4 to 5 nucleotides. This result confirms that this residue is responsible for the stacking of the RNA substrate in the catalytic cavity, as was predicted from the structure of Rrp44. Furthermore, we also show that a strain with a mutation in this residue has a growth defect and affects RNA processing and degradation. These results lead us to hypothesize that this residue has an important biological role. Molecular dynamics modeling of these Rrp44 mutants and the wild-type enzyme showed changes that extended beyond the mutated residues and helped to explain these results.

## Introduction

The exosome is an important protein complex involved in the maintenance of the correct levels of RNAs in the cell [1], [2]. The exosome processes some RNAs to shorter forms and participates in RNA degradation and surveillance. It is a highly conserved complex present in both eukaryotes and *Archaea*. The yeast exosome is a complex that consists of a core of ten proteins that are essential for viability and operates in the nucleus and in the cytoplasm [3]. Previous reports have shown that Rrp44 (also known as Dis3) is the only catalytically active nuclease in the yeast core exosome [4], [5] and plays a direct role in RNA surveillance contributing to the recognition and degradation of specific RNA targets [6]. Rrp44 is a member of the RNase II family of enzymes, since it has the RNB catalytic domain and is closely related to the 3′ hydrolytic exoribonucleases RNase II and RNase R from *Escherichia coli* [7], [8]. This family of proteins is ubiquitous and these enzymes can be found in all three domains of life. Members of this family are often essential for growth [9] and can be developmentally regulated [10]. In *Schizosaccharomyces pombe* both Dis3 and Dis3L2 are present but *Saccharomyces cerevisiae* only has Rrp44/Dis3 [11]. Dis3L2 preferentially degrades uridylated substrates and has defined a novel cytoplasmic decay pathway, independent of the exosome, which can degrade not only mRNAs but also other types of RNAs such as miRNAs [11]–[13]. In humans there are 3 homologues: Dis3, Dis3L1 and Dis3L2 [14], [15]. Mutations in Rrp44/Dis3 homologues cause abnormal chloroplast biogenesis [16], aberrant mitotic control, and human diseases such as multiple myeloma and Perlman syndrome [17]–[19].

The determination of the structure of RNase II provided an explanation for the dynamic mechanism of RNA degradation for this family of enzymes [7]. Rrp44 presents a similar modular organization to *E. coli* RNase II, consisting of a central nuclease domain (RNB) flanked by three oligonucleotide binding domains, two at the N-terminus and one at the C-terminus [7], [20]. In addition to these domains, yeast Rrp44 contains a conserved motif at the N-terminus that contains three conserved cysteine residues - CR3, and a highly conserved PIN domain [10], [20], [21]. We have recently shown that the three cysteines together with an histidine from the PIN domain affect the binding to the exosome [22]. We, and others have shown that in addition to the already known Rrp44 exonucleolytic activity, Rrp44 also has endonucleolytic activity associated with its PIN domain [23]–[25].

In order to ensure proper cellular RNA metabolism, the eukaryotic exosome needs to be tightly regulated. However, it is still unclear how the activity of the exosome is controlled. To address whether the exosome subunits or the exosome cofactors are responsible for the regulation of this macromolecular complex, we focused initially on the biochemical characterization of yeast Rrp44, and generated several mutants in conserved residues. A previous study of RNase II showed that a mutation in E542 resulted in a “super-enzyme” with a significant increase in ribonucleolytic activity [26]. In other studies it was also shown that Y253 in RNase II and Y324 in RNase R are critical in setting the size of the end-product in *in vitro* experiments [27], [28]. The crystal structure of yeast Rrp44 suggests that Y595 plays a similar role, but its function has not been experimentally addressed [20], [21]. To investigate whether the size of the end-product is important, we studied the role of the corresponding residues in yeast Rrp44.

## Materials and Methods

### Materials

Restriction enzymes, T4 DNA ligase and T4 polynucleotide kinase were purchased from Fermentas. Phusion DNA polymerase is from Finnzymes. Unlabeled oligonucleotide primers were synthesized by STAB Vida, Portugal.

### *E. coli* Strains

The *E. coli* strains used were XL1-Blue (*recA1 endA1 gyrA96 thi-1 hsdR17 supE44 relA1 lac* [F′ *proAB* lac*I^q^ZΔM15* Tn*10* (Tet^r^)]; Stratagene) for cloning experiments, and Rosetta(DE3)pLysS (F^−^ ompT hsdS_B_(r_B_^−^ m_B_^−^) gal dcm (DE3) pLysSRARE (Cam^R^); Novagen) for expression and purification of enzymes.

### Construction of *rrp44* Mutants by Site-Directed Mutagenesis

The point mutations Q892A and Y595A were introduced into *pGEX-RRP44* and *pRS415- RRP44* [24] by site-directed mutagenesis. The oligonucleotides used in this study were the following (base changes are indicated in lower case letters): forward oligonucleotide Q892Aa, 5′-CAGAAACGCCgcATTCGCCGGTAGGGCTaGCATAG-3′ and reverse oligonucleotide Q892Ab, 5′-ATGCtAGCCCTACCGGCGAATgcGGCGTTTCTGTG-3′; forward oligonucleotide Y595Aa, 5′-GGTACTTCTGTAgcTTTGGTcGAC-3′ and reverse oligonucleotide Y595Ab, 5′-GTCgACCAAAcgTACAGAAGTACC-3′; forward oligonucleotide G895Aa, 5′-CGCCCAATTCGCCGcTAGGGCtAGC-3′ and reverse oligonucleotide G895Ab, 5′-GCTaGCCCTAgCGGCGAATTGGGCG-3′; forward oligonucleotide G895Ea, 5′-CGCCCAATTCGCCGaaAGGGCtAGC-3′ and reverse oligonucleotide G895Eb, 5′- GCTaGCCCTttCGGCGAATTGGGCG-3′; forward oligonucleotide G895Qa, 5′- CGCCCAATTCGCCcaaAGGGCtAGC-3′ and reverse oligonucleotide G895Qb, 5′-GCTaGCCCTttgGGCGAATTGGGCG-3′. Each oligonucleotide also changes a restriction site. We screened putative mutants by restriction digestion and verified them by sequencing at STAB Vida, Portugal.

### Overexpression and Purification of Wild-Type Rrp44 and Mutants

Wild-type and mutated Rrp44 fused to GST were expressed in the Rosetta(DE3)pLysS *E. coli* strain. Cultures were grown in 2 liters TB medium supplemented with 200 mg ml^−1^ ampicillin and 34 mg ml^−1^ chloramphenicol at 30°C to an optical density at 600 nm (OD_600_) of 1.2 and then induced expression by addition of 0.2 mM IPTG and finally incubated overnight at 18°C. Cells were harvested by centrifugation and subsequently the pellets were frozen. Cells were thawed on ice, resuspended in 30 ml PBS buffer, pH 7.3, with 10 mM DTT and 1 mM PMSF, and lysed using the French Press. The crude extracts were treated with 5 units Benzonase (Sigma) for 30 min, and clarified by centrifugation at 10,000 g for 30-min. The clarified protein extract was loaded onto a GSTrap™ FF 1-ml column (GE healthcare), previously equilibrated in binding buffer. Proteins were eluted with 50 mM Tris-HCl, pH 8.2, 10 mM reduced glutathione. Fractions containing the purified protein were pooled and loaded onto a HiTrap™ Q FF column (GE Healthcare), previously equilibrated in 50 mM Tris-HCl, pH 8.2. Proteins were then eluted with a 0–1 M NaCl gradient in 50 mM Tris-HCl, pH 8.2, 1 mM DTT. The eluted protein was concentrated by centrifugation at 4°C with Vivaspin 500 Centrifugal Concentrators (Vivaspin). The protein concentrations were determined by spectrophotometry using a NanoDrop 1000 instrument (Alfagene) and 50% (v/v) glycerol and 1 mM DTT was added to the final fractions before storage at −20°C.

### Substrate Preparation

Exoribonucleolytic activity was assayed using different RNA oligoribonucleotides as substrates: a 30-mer oligoribonucleotide (5′-CCCGACACCAACCACUAAAAAAAAAAAAAA-3′) as a single-stranded substrate and a 30-mer oligoribonucleotide (5′-CCCGACACCAACCACUAAAAAAAAAAAAAA-3′) annealed to the complementary 16-mer oligoribonucleotide (5′-AGUGGUUGGUGUCGGG-3′) as a double-stranded substrate with a 3′-single-stranded extension. The 30-mer RNA oligoribonucleotide was labeled at the 5′-end with [γ-^32^ATP] and T4 polynucleotide kinase and further purified with MicroSpin G-25 columns (GE Healthcare) to remove the non-incorporated nucleotides. The hybridization between 16-mer and the radioactively labeled 30-mer oligomer was performed in a 1∶1.3 (mol∶mol) ratio in a buffer containing 10 mM Tris-HCl (pH 8.0) and 20 mM KCl. The mixture was then incubated at 95°C for 7 min and allowed to cool slowly to 4°C. Formation of the double stranded substrate was confirmed on a native polyacrylamide gel.

### RNase Assays

Exoribonuclease activity assays were performed in a final volume of 25 µl containing 20 nM of enzyme in the reaction buffer: 10 mM Tris-HCl pH 8, 75 mM KCl, 40 µM MgCl_2_ and 1 mM β-mercaptoethanol [4]. Control reactions were incubated with no enzyme added. Reactions were started by the addition of the substrate, directly or supplemented with 40 nM of the non-radioactive labeled oligonucleotide, and incubated at 37°C. Samples were withdrawn at the time points indicated in the figures, and the reaction was stopped by adding an equal volume of formamide-containing dye supplemented with 10 mM EDTA. Reaction products were resolved in 20% polyacrylamide, 8M urea gels. Gels were imaged using STORM 860 Molecular Imager scanner (GE Healthcare). The exoribonucleolytic activity of the enzymes was determined by quantifying the fraction of full-length RNA lost in distinct experiments using 20 nM of enzyme and 5′-radioactively labeled 30-mer oligoribonucleotide supplemented with 40 nM of non-radioactively labeled 30-mer oligoribonucleotide. Quantification was carried out using Image Quant software and each value obtained represents the mean of three independent assays.

### Comparative modelling of wild-type Rrp44 and Q892A and Y595A mutant proteins

Preliminary 3D-structural models of *S. cerevisiae* wild-type Rrp44 and mutant proteins Q892A and Y595A, were built by using standard comparative methods, the software DeepView [29] and the crystallographic structure of yeast Rrp44 D551N mutant protein bound to a 10-nucleotide poly(A) RNA strand (Protein Data Bank code 2VNU) [20]. In order to obtain uninterrupted theoretical structures of residues 252–1001 of Rrp44, the template set was partially enriched with segments from the apo form of Rrp44 D551N (Protein Data Bank code 2WP8) [30]. Only fragments not affected by conformational changes between apo and bound states were used for this purpose [20],[30]. Overall structural quality of all rendered models was checked using the analysis programs Anolea, Gromos and Verify3D provided by SWISS-MODEL server (http://swissmodel.expasy.org) [31]–[33].

### Modelling of Rrp44 molecular interactions to single-stranded poly(A) RNA

In order to model 3D-structural interaction of wild-type Rrp44, and mutants Q892A and Y595A with a single-stranded poly(A) RNA molecule (RA1–RA10), preliminary structures were set up by translating the RNA ligand coordinates from the PDB structure 2VNU [20] over each comparative model. The recently published structure of the Rrp44 D171N/D551N mutant in complex with poly(U) RNA at the RNB domain [21] was used for comparative purposes. To obtain a set of refined models, a standard molecular dynamics protocol of 6 ns was performed for every Rrp44-ssRNA system, essentially as described elsewhere [26], [27]. All the energy minimizations and molecular dynamics simulations were performed with the PMEMD module of the AMBER11 package [34], using molecular mechanics parameters in *parm99* [34] plus *parm99SBild* modifications [35] and *ff99bsc0* [36] sets. For every Rrp44-ssRNA system, one minimized average structure was taken from the final most stable segment of simulation, as representative of the whole ensemble. Root-mean-square-deviation (RMSD) trace analyses of the peptide-backbone in the RNB domain (residues 475–911), and the phosphate-backbone in nucleotides RA1–RA5 were determined with the program PTRAJ from the same Amber suite. All RMSD point values were calculated from the initial conformation of the three modelled Rrp44-ssRNA systems. All structures were manipulated and visually rendered using PyMOL.

### Yeast Complementation Assay

An *rrp44Δ* strain complemented by wild-type *RRP44* on a plasmid with a *URA3* selectable marker was transformed with *LEU2* plasmids encoding each of the point mutations. To test whether these mutations were essential for growth, the transformants were serially diluted by a factor of five and spotted onto plates containing 5-fluoro-orotic acid (5-FOA) or control plates (SC-LEU-URA). Growth on 5-FOA indicates that the mutated Rrp44 can carry out the essential function of the exosome.

### *his3-nonstop* Growth Assay

To investigate whether the mutations of Rrp44 disrupt exosome-mediated mRNA decay we used the two viable Rrp44 mutations in a *his3-nonstop* growth assay. *ski7Δ* [Vector, *LEU2*], *rrp44Δ* [*RRP44, LEU2*], *rrp44Δ* [*rrp44-Q892A, LEU2*] and *rrp44Δ* [*rrp44-Y595A, LEU2*] strains were transformed with a mutant *his3* gene lacking a stop codon, on a plasmid with a *URA3* marker. The *ski7Δ* strain was included because it is known to stabilize the his3-nonstop mRNA. The transformants were selected on SC-URA-LEU, and then serially diluted and spotted on SC-HIS and SC-URA-LEU plates. Growth on SC-HIS plates indicates a nonstop mRNA defect.

### *xrn1Δ* synthetic lethality assay

To further investigate whether the mutations of Rrp44 disrupt exosome-mediated mRNA decay we also tested for synthetic lethality with *xrn1Δ*, essentially as previously described [37]. Briefly, plasmids encoding the rrp44 point mutations were transformed into the *rrp44Δ xrn1Δ* strain containing a *URA3* plasmid encoding wild-type Rrp44. Transformants were grown in SC-LEU-URA and serially diluted in 96-well plates by a factor of five and spotted onto media containing 5-FOA. In the absence of Xrn1, the exonuclease activity of Rrp44 is solely responsible for bulk mRNA degradation, and therefore growth on 5-FOA indicates that the Rrp44 allele on the *LEU2* plasmid is capable of mRNA degradation.

### Northern Blot Analysis

The *RRP44*, *rrp44-D171A*, *rrp44-D551N*, *rrp6Δ*, *rrp44-Q892A*, *rrp44-Y595A* strains were grown in YPD, total RNA was isolated, resolved on polyacrylamide gels, and probed with 5′ ^32^P-labelled oligonucleotides for 5.8S processing defects (5′-TTTCGCTGCGTTCTTCATC-3′), 5′ ETS degradation defects (5′-CGAACGACAAGCCTACTCG-3′), and as a loading control the RNA subunit of the signal recognition particle (SRP - 5′-GTCTAGCCGCGAGGAAGG-3′).

## Results

### Mutation of Q892 enhances Rrp44 activity

**Figure 1:**
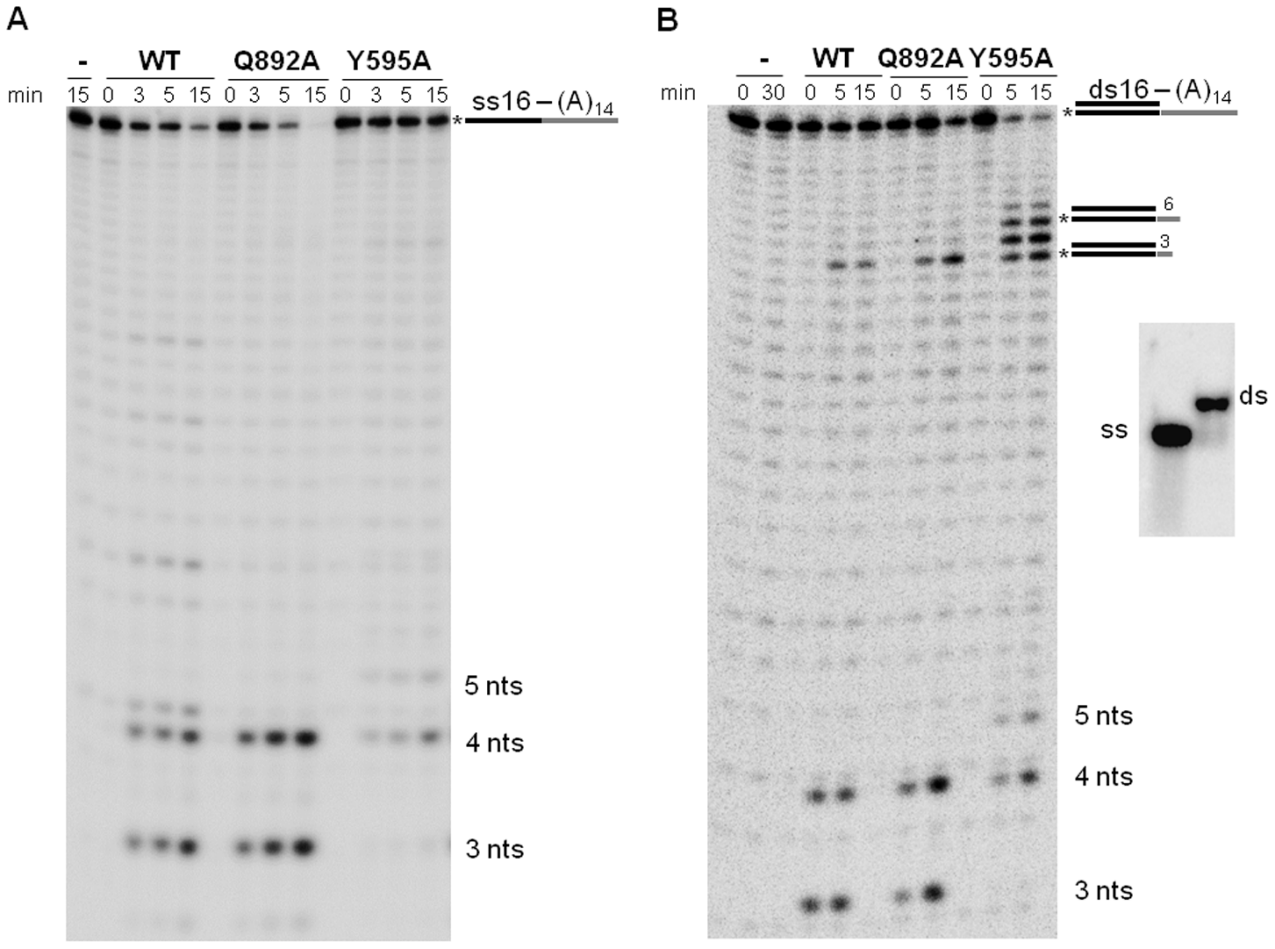
Exoribonuclease activity of Rrp44 and mutant versions of Rrp44. Activity assays were performed as described in Materials and Methods using 20 nM of enzyme (WT, Q892A and Y595A) and different substrates. Reactions were stopped and samples were taken at the time-points indicated. Length of substrates and degradation products are indicated in the figure. In panel A, 30-mer 5′-radioactively labeled RNA substrate (5′-CCCGACACCAACCACUAAAAAAAAAAAAAA-3′) was supplemented with 40 nM of substrate nonlabeled. In panel B, 30-mer 5′-radioactively labeled RNA substrate (5′-CCCGACACCAACCACUAAAAAAAAAAAAAA-3′) was annealed with a RNA oligonucleotide (5′-AGUGGUUGGUGUCGGG-3′). Figure on the right side depicts the control of the annealing of the double-stranded (ds) substrate on a native polyacrylamide gel.

A previous study of *E. coli* RNase II showed that a mutation in E542 resulted in a “super-enzyme” with a significant increase in ribonucleolytic activity [26]. Analysis of both structural and sequence alignments showed that G895 residue in yeast Rrp44 and E542 in *E. coli* RNase II occupy the same position (Figure S1, Figure S2). Thus we chose to mutate G895. Since G895 is not universally conserved in Rrp44 orthologs, we also targeted Q892. Q892 is more broadly conserved, one turn away from G895 on the same alpha helix and chemically more similar to E542 (Figure S1). Therefore we hypothesized that Rrp44 Q892 might functionally replace RNase II E542. Thus, mutations G895A, G895E, G895Q, and Q892A in yeast Rrp44 could be viable candidates to observe similar effects than those revealed by the RNase II E542A mutant. In order to analyze the role of these residues we purified the proteins containing these mutations and compared their activities to the purified wild-type Rrp44 protein by performing exoribonuclease assays using a 30-mer ssRNA substrate (Figure 1; Figure S3A, B). As previously demonstrated, the wild-type Rrp44 enzyme was able to degrade the 30-mer ssRNA substrate to a final major product of 4 nts [20]. Two of the mutations, G895A and G895Q, showed a reduction of the ribonucleolytic activity when compared to the wild-type Rrp44 protein (Figure S3A). The G895E mutant presented activity very similar to that of the wild-type enzyme. Most interestingly, the Q892A mutant had a reproducible increase in catalytic activity (Figure 1; Figure S3A). Quantitating the fraction of substrate lost showed that the activity of the Q892A mutant enzyme was increased more than 2 fold when compared to its wild-type counterpart (0.56 nmol substrate digested per min per nmol enzyme *versus* 0.25 nmol.min^−1^.nmol^−1^; Figure S4).

Similarly to wild-type, Q892A mutant was able to degrade a double-stranded substrate with a 3′ single-stranded extension (ds16–30) to a final major product of 4 nts (Figure 1B).

### Mutation of Y595 alters the Rrp44 end-product

Members of the RNase II family each produce a distinct end-product, but the length of this product differs between family members. For example, RNase II and Rrp44 produce 4 nts end products, but RNase R continues degradation to a 2 nts end product. The biological significance of these differences is not understood, but the end product can be changed by mutating a conserved Y residue. Previous reports showed that a Y253A mutation in *E. coli* RNase II changed the end product from 4 to 10 nts, while the equivalent change in *E. coli* RNase R changed it from 2 to 5 nts [27], [28]. The role of this residue in setting the end product is thought to be explained by its stabilization of the 3′-end of the RNA molecule in the catalytic site. One of the aims of our work was to determine the specific role of the equivalent residue in yeast Rrp44. This residue is highly conserved in the RNase II family of enzymes and by analysis of the sequence alignment of RNase II, RNase R and Rrp44 proteins we and others determined that this specific residue is equivalent to Y595 in yeast Rrp44 [20], [28]. We mutated the aromatic residue Y595 in Rrp44 into an alanine, purified the mutant protein, and assessed its enzymatic activity (Figure 1; Figure S3B). Our results demonstrate that this mutation altered the size of the final product of degradation of a generic substrate. As shown in Figure 1 the major end-product of Rrp44 is 4 nts in length, while the Y595A mutant produced a major final product of 5 nts. Our data also suggest that this mutation resulted in a decrease in catalytic activity (0.25 and 0.05 nmol substrate digested per min per nmol enzyme for the wild-type and Y595A mutant, respectively; Figure S4).

When a double-stranded substrate with a 3′ single-stranded extension was tested, the same end-product was rendered (Figure 1B). Comparing with wild-type, the appearance of the final product is slower in the Y595A mutant and accumulated 19–22 nt oligomers which shows that this mutant stalls 3–6 nt before reaching double-stranded structure.

### Theoretical structural insights of Rrp44 interaction to RNA at the RNB domain

In order to understand the molecular basis for the results obtained, we have generated theoretical structural models. Our models are based on x-ray structures of Rrp44 either in the RNA bound [20] or apo [30] form. Importantly, all available experimentally determined structures are of a catalytically inactive form (D551N), while our models are of the active enzyme. After six nanoseconds of free molecular dynamics, representative Rrp44 models for the wild-type, Y595A, and Q892A enzymes showed no relevant differences in their secondary and tertiary structures. According to RMSD data, only the ssRNA bound Q892A mutant shows significant change in its peptide backbone (Figure S5). However, a detailed inspection of the catalytic cleft provided a set of interesting local variations that modify ssRNA binding patterns in the three models.

#### Wild-type model

**Figure 2:**
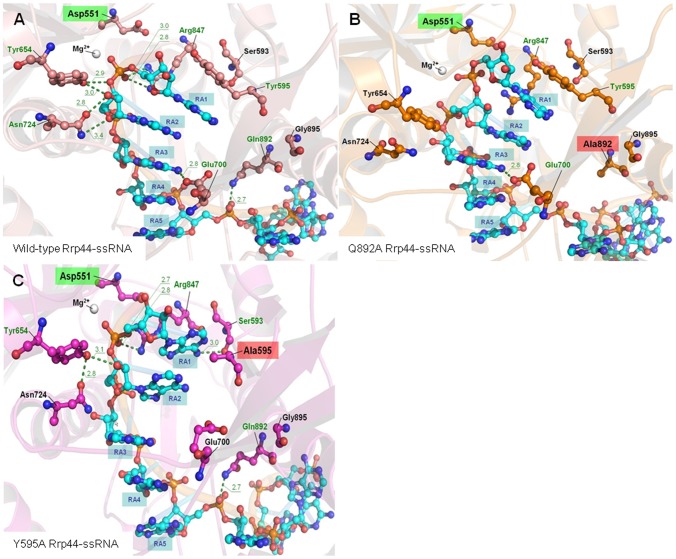
Theoretical catalytic site of Rrp44 RNB domain in *S. cerevisiae*. Wild-type (panel A) and mutants Q892A (panel B) and Y595A (panel C), in complex with one single strand poly(A) RNA molecule. Relative locations of some functionally relevant residues described in the text, depicted over every modeled protein's 3D structure. Also layed out inside color text boxes, *in silico* reverted mutation Asp551 (green), Q892A and Y595A mutations (red), and nucleotide bases (blue) starting from RA1 (outgoing). Hypothetical hydrogen-bond mediated interactions, residue tags and annotations of corresponding point distances (in Angstroms, measured over every final representative model) depicted in green color. Other important residues, hypothetically invariant among modeled proteins, not shown for clarity.

The modeled structure of wild-type Rrp44 suggests that the general pattern of interactions between its RNB domain and the five most 3′ ssRNA nucleotides (RA1–RA5) remains functionally identical to the one in the D551N RNA-bound crystal structures, but our model suggest several additional contacts not present in the crystal structure of the D551N mutant enzyme (Figure 2A) [20], [21]. Specifically, in our model Y654 contributes to binding of the phosphate group of the outgoing nucleotide (RA1), as well as the 2′-OH moiety of nucleotide RA2. Interestingly, the equivalent RNase II residue (Y313) occupies a similar position and was suggested to contribute to catalysis by facilitating protonation of the 3′OH of RA2 [13]. The contribution of R847 to binding the phosphate group of RA1 also seems to be reinforced in the model. Additionally, our model indicates that residue N724 can form hydrogen-bonds with the 2′-OH group and the ribose ring of RA2, contributing to stability of the nucleotide preceding the bond to be broken. Finally, the side chain of Q892 is hydrogen-bonded to nucleotide RA5, contributing to stabilize the poly(A) chain at this spot. Overall, comparison of our model of the wild-type enzyme to the experimentally determined structures of the D551N mutant suggests a strengthening of the interactions between Rrp44 and nucleotides RA1, RA2 and RA5. Clarifying the importance of these interactions will require further structural, biochemical and genetic experiments.

#### Q892A mutant model

We analysed Q892 hypothesizing that it might function similarly to E542 of RNase II. However, unlike the hydrogen bonding of E542 to the base of RA1, the amide group of Q892 forms a hydrogen-bond to the backbone of nucleotide RA5, which cannot be formed upon mutating this residues to A (Figure 2B). However, our model suggests additional changes in the catalytic cleft, resulting in weaker and more labile substrate binding than in the wild-type model and D551N structures [20], [21]. For example, the side chain of Y654 has moved away from the ssRNA and now could be too far away (beyond 4.0 Angstroms) to form a strong hydrogen-bond. In addition, the side chain orientation of R847 resembles that seen in the D551N structure and its electrostatic interaction with the phosphate-backbone of RA1 nucleotide could be relaxed [20], [21]. However, despite that the Q892A mutation could weaken some direct interactions, the continuous self-stacking organization of nucleotides RA1–RA5 to Y595 is maintained. Therefore, the model of the Q892A protein is consistent with its near wild-type ability to bind ssRNA in a catalytically active form. The increased lability may explain the enhanced activity of this mutant.

#### Y595A mutant model

Analyses of the catalytic cleft of the Y595A model suggests that this mutation distorts the continuity of self-stacking interaction between the five most 3′ ssRNA nucleotides (RA1–RA5; Figure 2C). Although nucleotides RA5-RA3 are organized similarly to the wild-type enzyme, stacking between RA3 and RA2 is disrupted while RA1 remains stacked on RA2. Other hypothetical changes in the catalytic cleft suggest that a general easing of interactions with nucleotides RA2–RA5 might take place. For example, in contrast to the wild-type model and the D551N mutant structures [20], [21], the side chain of E700 in Y595A mutant would be exposed to solvent, unable to stabilize the adenine base in nucleotide RA3. At the same time, N724 does not seem to directly contact ssRNA in this mutant, but it still contributes to the position of Y654 which is bound to the 2′-OH moiety of RA2. These alterations in continuity of π-stacked interactions combined with the easing of strong interactions with RA2 and RA3 could result in an increase in the lability of nucleotides RA2–RA5, reduce the stability of substrates smaller than 6 nucleotides, and consequently hinder catalysis with such fragments. However, other relevant changes in the local environment of the outgoing nucleotide (RA1) indicate that its tight binding would be preserved during the catalytic events. For example, rather than being exposed to solvent, the base of RA1 could be interacting with the backbone carbonyl oxygen of S593, and a stronger electrostatic interaction (in terms of distance to phosphate group) of R847 with the phosphate in RA1 could also be possible.

### Assessing the functions *in vivo* of the Q892 and Y595 residues

**Figure 3:**
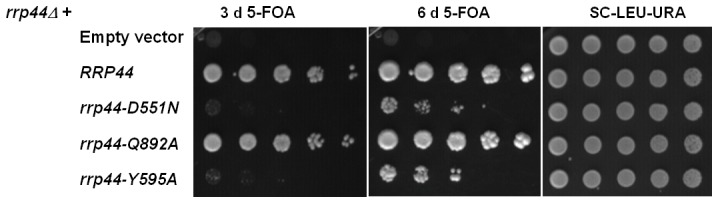
The Y595 residue is important for exosome function. A *rrp44Δ* strain complemented by full-length *RRP44* on a plasmid with a *URA3* marker was transformed with *LEU2* plasmids encoding each of the mutations. Growth on 5-fluoroorotic acid (5FOA) indicates that *rrp44-Q892A* can carry out the essential function of the exosome. Mutation to alanine of the tyrosine 595 residue reduces growth.

In order to test whether the changes in activity affect the function of the exosome *in vivo*, we performed growth assays with the *rrp44-Q892A* and *rrp44-Y595A* mutants. An *rrp44Δ* strain complemented with wild-type *RRP44* on a plasmid with a *URA3* selectable marker was transformed with *LEU2* plasmids encoding each of the two point mutations, Q892A and Y595A. The empty vector and a plasmid with the wild-type *RRP44* gene were used as negative and positive controls, respectively. Transformants were spotted onto plates containing 5-fluoro-orotic acid (5-FOA), which kills cells that contain the *URA3* plasmid. Growth on 5-FOA indicates that the *URA3-RRP44* plasmid was lost and that the mutated Rrp44 encoded on the *LEU2* plasmid can carry out the essential function of the exosome. As compared to the wild-type *RRP44*, the mutant *rrp44*-*Q892A* strain does not have an obvious growth defect, suggesting that this mutation does not affect the essential function of the exosome *in vivo* (Figure 3). In contrast, the strain containing the *rrp44*-*Y595A* version is viable, but grows significantly slower than the wild-type strain. Instead the growth rate of the *rrp44-Y595A* strain was comparable to that of the catalytically dead *rrp44-D551N* mutant. The growth defect of the *rrp44*-*Y595A* mutant suggests an important biological role for the Y595 residue that may be related to the alteration of the end-product induced by this mutation.

### Q892A and Y595A mutations do not affect mRNA decay

**Figure 4:**
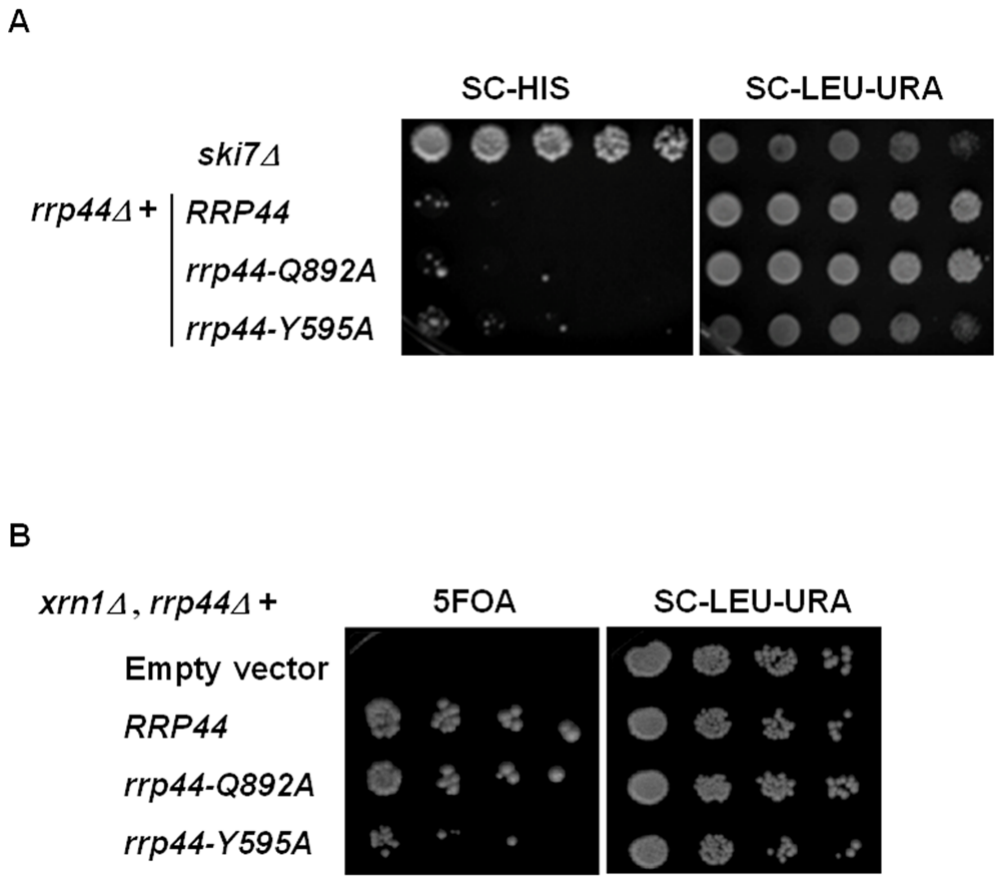
*rrp44-Y595A* and *rrp44-Q892A* do not have a defect in mRNA decay. **A**. A *ski7Δ* strain [Vector, *LEU2*], *rrp44Δ* [*RRP44, LEU2*], *rrp44Δ* [*rrp44-Q892A, LEU2*] and *rrp44Δ* [*rrp44-Q892A, LEU2*] were transformed with a mutant *his3* gene on a plasmid with a *URA3* marker. The transformants were selected on SC-URA-LEU. Growth on SC-HIS indicates that the *his3-nonstop* mRNA is stabilized while failure to grow indicates normal nonstop mRNA decay. SC-LEU-URA was used as control. **B**. An *xrn1Δ rrp44Δ* strain containing an *RRP44*, *URA3* plasmid was transformed with *LEU2* plasmids that encoded either no Rrp44, wild-type Rrp44, Rrp44-Q892A or Rrp44-Y595A. Growth on plates containing 5-FOA indicates that the resulting transformants are viable upon loss of the *URA3* plasmid, and thus that the encoded Rrp44 is capable of degrading mRNA. SC-LEU-URA was used as control.

We used two assays to investigate the effect of the Rrp44 mutations on mRNA degradation. First, inactivation of the cytoplasmic exosome results in the stabilization of transcripts that lack stop codons [38], [39]. To investigate whether the Q892A or Y595A mutations affect exosome-mediated mRNA decay, we analyzed the expression of a *his3-nonstop* reporter [39]. The aberrant *his3-nonstop* mRNA is normally rapidly degraded by the cytoplasmic exosome. This results in low levels of His3 protein, and a failure to grow in the absence of histidine. A defect in nonstop mRNA decay results in growth on media lacking histidine due to stabilization of the *his3-nonstop* mRNA. As previously described, deletion of the gene encoding the cytoplasmic exosome cofactor Ski7 allowed for growth on media lacking histidine; an important control showing that the *his3-nonstop* mRNA transcript was stabilized in this strain [39]. In contrast, the wild-type strain and both mutants of *RRP44* (*rrp44-Q892A* and *rrp44-Y595A*) failed to grow on the plate without histidine (Figure 4A). Thus, this result indicates that the *his3-nonstop* mRNA was unstable and that none of the mutations in *rrp44* affect *nonstop* mRNA degradation.

In a second assay for mRNA degradation we investigated synthetic lethality with *xrn1Δ*. Unlike nonstop mRNAs, the degradation of normal mRNAs is mostly carried out by Xrn1 with Rrp44 making a smaller contribution. Therefore, mutations in Rrp44 do not affect the decay of normal mRNAs, but because of the redundancy between the pathways the combination of *xrn1Δ* and *rrp44* mutations that interfere with normal mRNA decay is synthetically lethal. Figure 4B shows that the *xrn1Δ rrp44-Q892A* double mutant grows like the *xrn1Δ* single mutant, and thus there is no synthetic lethality. However, the *xrn1Δ rrp44-Y595A* mutant grows slower than the *xrn1Δ* single mutant, but this could be explained by the slow growth of the *rrp44-Y595A* single mutant and not by synthetic lethality. The absence of genetic interactions suggests that both *RRP44* alleles are functional for bulk mRNA degradation.Y595 is important for RNA-processing and degradation.

The exosome carries out both RNA-processing and RNA degradation reactions. To determine if Rrp44 residues Q892 and Y595 are required for these roles of the nuclear exosome, we isolated RNA from each mutant strain and analyzed it by Northern blotting for aberrant processing of the 7S pre-RNA to 5.8S rRNA, and degradation of the 5′-external transcribed spacer (5′-ETS). These RNA species are products of the 35S polycistronic precursor rRNA.

**Figure 5:**
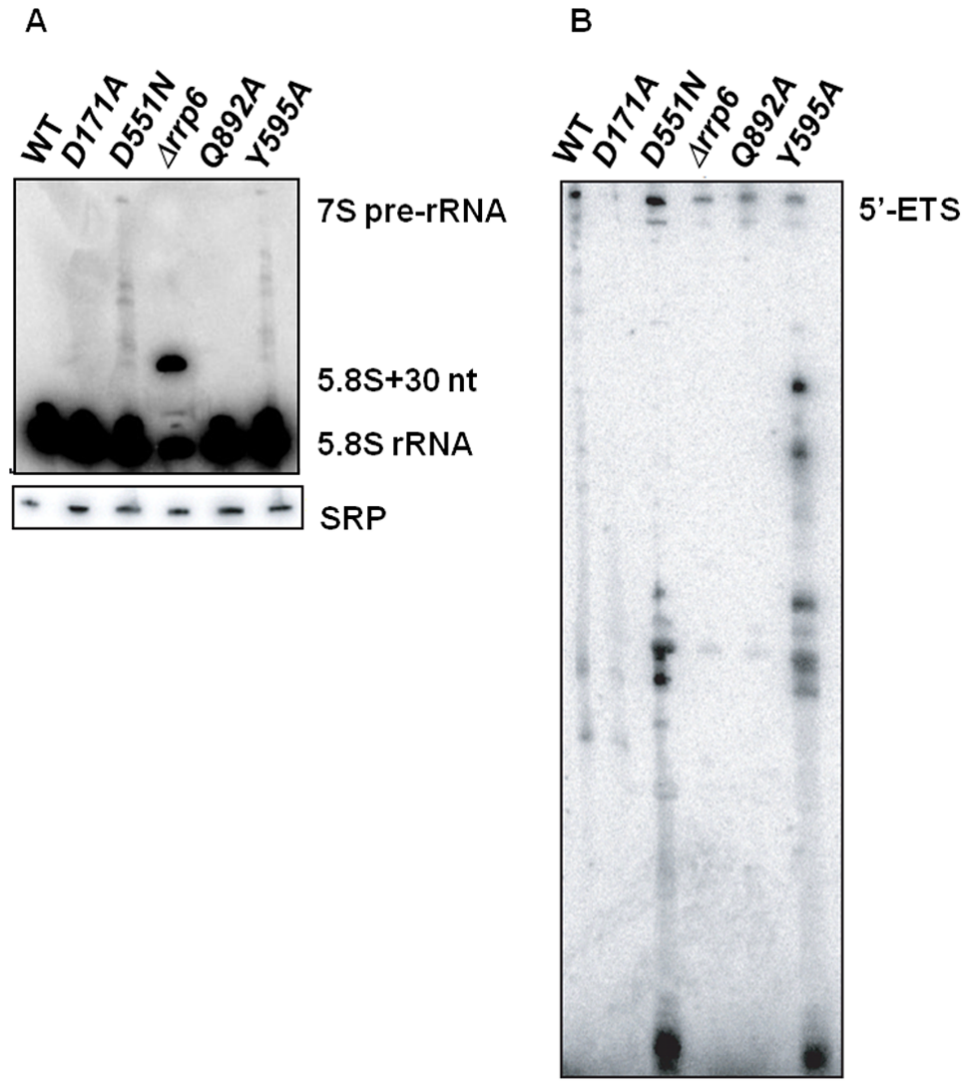
Y595 residue is required for both RNA-processing and degradation. The indicated Rrp44 proteins were expressed in a *rrp44Δ* yeast strain. RNA was isolated from cultures grown in rich medium and analyzed by northern blotting with probes that hybridize to the 5′ ETS region (panel A), 5.8S rRNA (panel B) and the RNA subunit of the signal recognition particle (SRP).

The 5.8S rRNA is generated from a 7S precursor by the exosome and Mtr4 [40]–[42]. It was already reported that 7S-processing intermediates accumulated as a result of the D551N mutation in the RNB domain but not from the D171A mutation in the PIN domain [23], [24]. In contrast, the *rrp6Δ* strain does not lead to the accumulation of these species, but instead causes the accumulation of a distinct RNA species that is 30 nucleotides longer than the normal 5.8S rRNA [42]. Similar to the D551N mutant, the *rrp44-Y595A* strain causes the accumulation of heterogeneous species that range in length between 7S pre-RNA and 5.8S rRNA. This indicates that residue Y595 contributes to the processing of the 5.8S rRNA (Figure 5A).

The 5′-ETS is a by-product of rRNA processing which is degraded by the exosome after cleavage from 35S pre-RNA [40]. Northern blotting using a probe for the 5′-ETS revealed the accumulation of intermediates smaller than 5′ETS in *rrp44-Y595A* and *rrp44-D551N*, but not in *rrp44-D171A* and wild-type *RRP44* (Figure 5B). This result is in agreement with previous findings obtained for the *rrp44-D551N* and *rrp44-D171A* strains [23]–[25]. However, the size of the intermediates accumulated in *rrp44-Y595A* strain is different from the ones that accumulate in *rrp44-D551N* strain. In addition to the involvement in the maturation of 7S pre-rRNA, the Y595 residue appears to play an important role in the degradation of the excised 5′-ETS pre-rRNA region.

The *rrp44-Q892A* mutant strain does not show any specific RNA degradation or processing defects on 7S pre-rRNA, 5.8S rRNA and 5′ ETS, since it always showed the same pattern of degradation as wild-type *RRP44* and *rrp44-D171A* (Figure 5A, B). This result is not surprising, since this mutation did not cause a growth defect phenotype.

## Discussion

### Mutations in Rrp44 change exosome activity and the size of the end-product

We performed multiple sequence alignments of RNase II homologues in order to find which Rrp44 residues corresponded to *E. coli* RNase II residues that were shown to be important for activity and size of the end-product. Both structural and sequence alignments showed that G895 in yeast Rrp44 is the residue that occupies the same position as E542 in *E. coli* RNase II. Previous studies reported that the carboxylic group of E542 is in close proximity to the nitrogen atoms of the leaving nucleotide, and the establishment of one or more hydrogen-bonds between them could facilitate the elimination of this nucleotide after cleavage of the RNA substrate by RNase II [26]. Although G895 is in the same position, it clearly could not carry out this proposed function, and consistent with this, our results demonstrate that mutations in the G895 residue do not affect the protein catalytic activity. Instead of the G or E found in yeast and *E. coli*, many vertebrate Rrp44's have a Q in this position, and all eukaryotic Rrp44's have a Q one turn of the helix away (i.e. Q892 in yeast). These Q residues are a more likely candidate to carry out the same role ascribed to RNase II E542. Substitution of Q892 by alanine in yeast Rrp44 (Q892A mutation) led to an increase in ribonucleolytic activity, similar to what happens with the *E. coli* RNase II E542A mutant. However, the increase in activity of the Rrp44 Q892A mutant was much more moderate. Interestingly, in our modeling this Q892 was hydrogen bonded to the phosphate between RA5 and RA6 instead of to the base of RA1, which may explain why Q892A in Rrp44 and E542A in RNase II have such different effect on activity.

Here we report that when Y595 residue is substituted by an alanine, the *in vitro* end-product changed from 4 nts to 5 nts. These data suggest that the role of this aromatic residue in setting the protein end-product is highly conserved. Moreover, this replacement also resulted in a pronounced decrease in the protein catalytic activity *in vitro* and less efficiency in the digestion of double-stranded structures. The *in vitro* results may explain the negative effect on exosome function *in vivo*, since this mutation does not affect the stability of the protein (data not shown).

### Theoretical structural responses and ribonucleolytic activity

Both substitutions seem to modify the relative organization of nucleotides RA2–RA5 in the models, making it more labile than what is expected in the wild-type model presented here. Although further structural modifications induced by Q892A mutation is possible, a faster processing capacity could explain the experimentally determined two-fold higher activity. Nevertheless, this work cannot dismiss the possibility that other changes in the catalytic complex could in turn modify the energetic barriers, facilitating the reaction and processing. From an evolutionary perspective, it is remarkable that the Rrp44 RNB domain has a wide capacity to accommodate mutations in the catalytic cleft. Interestingly, by performing undetermined global adaptive conformational changes, as in the Q892A model (Figure S5), the whole protein is able to remain functionally competent despite possible solvent displacement effects or changes in direct binding interactions. In relation to the latter hypothesis, our experimental results on G895A, G895Q, and G895E are also extraordinarily meaningful, as they show that this position is minimally related or irrelevant to the structure of protein-RNA-solvent complex. Nevertheless, more extensive studies involving the other domains of Rrp44 are still required to generate deeper insights in the fine structure and dynamics of the RNB-RNA complex during catalysis. In the Y595A model, an increased lability of nucleotides RA2–RA5 also seem to induce unsteady bound conformations in ssRNA substrates smaller than 6 nucleotides, hindering catalysis.

### Y595 residue has an important biological role

We showed for the first time that an alteration in Y595 residue changes the length of the end-product, decreases the activity of the enzyme and affects the ability to degrade secondary structures. Moreover, an alteration in this residue was shown to have implications for the cell, since the strain carrying the Y595A mutation has an effect in growth phenotype. We suggest that all 3 defects in Rrp44 exonucleolytic activity can contribute to the growth defect observed in this mutant.

The growth phenotype caused by this mutation also leads us to hypothesize that the alteration of the size of the end-product leads to the accumulation of degradation fragments in the cell. In *E. coli*, oligoribonuclease (*orn* gene) is required for the complete degradation of mRNA to mononucleotides and this process is required for cell viability [43], [44]. Oligoribonuclease has been described as a single-stranded specific RNase that has strong affinity for small RNA fragments (2–5 nts), with a 5-mer oligoribonucleotide being its preferred substrate [43], [44]. The 5 nucleotide end-product left by the Rrp44-Y595A mutant may not be a suitable substrate for degradation by an unknown yeast oligoribonuclease, and subsequently, the accumulation of small RNA fragments might have toxic effects for the cell and impair cellular growth.

## Conclusion

Overall, this study sheds light on the model previously proposed for Rrp44 activity and mode of action, and allows for an important step forward in the understanding of the mechanism of RNA processing and degradation by the exosome. Based on sequence and structure alignment we predicted that RNase II E542 and Rrp44 Q892 would also have similar roles, but biochemical analysis showed that Q892A had a much smaller effect on activity than expected from the RNase II data, which can be explained by our modeling suggesting that these two residues have different roles. Interestingly, Rrp44 Y595 and RNase II Y253 have very similar roles in the two enzymes, being critical residues in setting the smallest degradation product. Furthermore, we also show that a strain with a mutation in this residue has a growth defect and affects RNA processing and degradation.

## Supporting Information

Figure S1 **Partial multiple sequence alignment of Rrp44 homologues.** The full length sequences of Rrp44 homologues were aligned using ClustalW (http://www.ebi.ac.uk/Tools/msa/clustalw2/) and boxshade (http://www.ch.embnet.org/software/BOX_form.html) with default settings. This figure shows only a small part of the alignment, in yeast Rrp44 Q892-G895 region. Positions of yeast Q892 and conserved G895 are indicated. Red: residues identical in at least half of the sequences. Blue: residues similar in at least half of the sequences.
(TIF)

Figure S2 **Partial multiple structural alignment of Rrp44 homologues.** Superposition of *E. coli* RNase II (PDB ID 2IX1) [7] and *S. cerevisiae* Rrp44 (PDB ID 2VNU) [20] indicates that residue *E.coli* E542 and *S. cerevisiae* G895 residues occupy the same position in the space. Structures were rendered using PyMOL.
(TIF)

Figure S3 **Exoribonuclease activity of Rrp44 and the different mutants.** (A) Activity assays were performed as described in Materials and Methods using 20 nM of enzyme and 30-mer 5′-radioactively labelled RNA substrate (5′-CCCGACACCAACCACUAAAAAAAAAAAAAA-3′). Reactions were stopped and samples were taken at the time-points indicated. Length of substrates and degradation products are indicated in the figure. (B) SDS-PAGE analysis of wild-type and Rrp44 mutants. Equal amounts of mutant proteins were loaded into each lane; the gel was stained with Coomassie blue for detection.
(TIF)

Figure S4 **Exoribonuclease activity of wild-type Rrp44, Q892A and Y595A mutant.** Exoribonucleolytic activity of the enzymes was determined by quantifying the fraction of full-length RNA lost using 20 nM of enzyme (WT, Q892A and Y595A) and 5′-radioactively labeled 30-mer oligoribonucleotide supplemented with 40 nM of non-radioactively (5′-CCCGACACCAACCACUAAAAAAAAAAAAAA-3′). Graphs depict the degradation of substrate at different time points calculated using Image Quant. Each value represents the average of three independent assays.
(TIF)

Figure S5 **General structural deviations of RNB-ssRNA theoretical models from yeast Rrp44 wild-type and mutants.** RMSD temporal profiles of RNB domain peptide-backbones (upper panel) and five most 3′ ssRNA nucleotides (RA1–RA5) phosphate-backbone (lower panel) traces. RMSD point values and running averages (over 20) plotted in colours red (wild-type), blue (Q892A) and green (Y595A). RMSD measurements to initial conformations, revealed some interesting differential responses during the last segment of the simulations (Table S1), highlighted in soft yellow colour. Data from Rrp44 Q892A suggest that this mutation might be able to induce significant variability in the peptide-backbone of the RNB domain, in comparison to the wild-type and Y595A mutant. On the other hand, little variations seem to be affecting the phosphate-backbone structure of ssRNA within the catalytic cleft (nucleotides RA1–RA5) in the three phenotypes, although subtle differences could be altering it when bound to Y595A mutant.
(TIF)

Table S1 **Average RMSD values.** Average RMSD values obtained from the last nanosecond of free molecular dynamics trajectories of the three yeast Rrp44-ssRNA interaction models calculated. Data correspond to peptide-backbone trace of RNB domains and nucleotides RA1–RA5 in RNA bound molecules respectively.
(DOCX)
