# Correction: Modulating the RNA Processing and Decay by the Exosome: Altering Rrp44/Dis3 Activity and End-Product

**DOI:** 10.1371/journal.pone.0136810

**Published:** 2015-08-21

**Authors:** Filipa P. Reis, Ana Barbas, A. A. Klauer-King, Borislava Tsanova, Daneen Schaeffer, Eduardo López-Viñas, Paulino Gómez-Puertas, Ambro van Hoof, Cecília M. Arraiano

There are errors in the Funding section. The correct funding information is as follows: F.P.R. was a recipient of a Fundação para a Ciência e a Tecnologia (FCT) Ph.D. fellowship and A.B. was a recipient of a FCT PostDoc. fellowship. This work was supported by grants from FCT, Portugal (including grants PTDC/QUI-BIQ/111757/2009 and PEst-OE/EQB/LA0004/2011) and by grant FP7-KBBE-2011-1-289326 from European Commission. Work in the A.v.H. laboratory was funded by grants from National Institutes of Health [R01GM099790], the Welch foundation [AU-1773] and an EMBO short term fellowship to [D.S.]. Work at the P.G-P. laboratory was supported by: the Spanish Ministerio de Ciencia e Innovación [grants SAF2007-61926, IPT2011-0964-900000 and SAF2011-13156-E] and the European Commission [grants FP7 HEALTH-F3-2009-223431 (EU project “Divinocell”) and FP7 HEALTH-2011-278603 (EU project “Dorian”)]. Support from the “Fundación Ramón Areces” and the Centro de Computación Científica CCC-UAM” for computational support is also acknowledged. Work at Biomol-Informatics was partially financed by the European Social Fund. The funders had no role in study design, data collection and analysis, decision to publish, or preparation of the manuscript.
