# Supplementary figures and images for: Modulating the RNA Processing and Decay by the Exosome: Altering Rrp44/Dis3 Activity and End-Product
Source: PLoS One. 2013 Nov 12;8(11):e76504. doi: 10.1371/journal.pone.0076504 (PMC3827031; doi:10.1371/journal.pone.0076504)

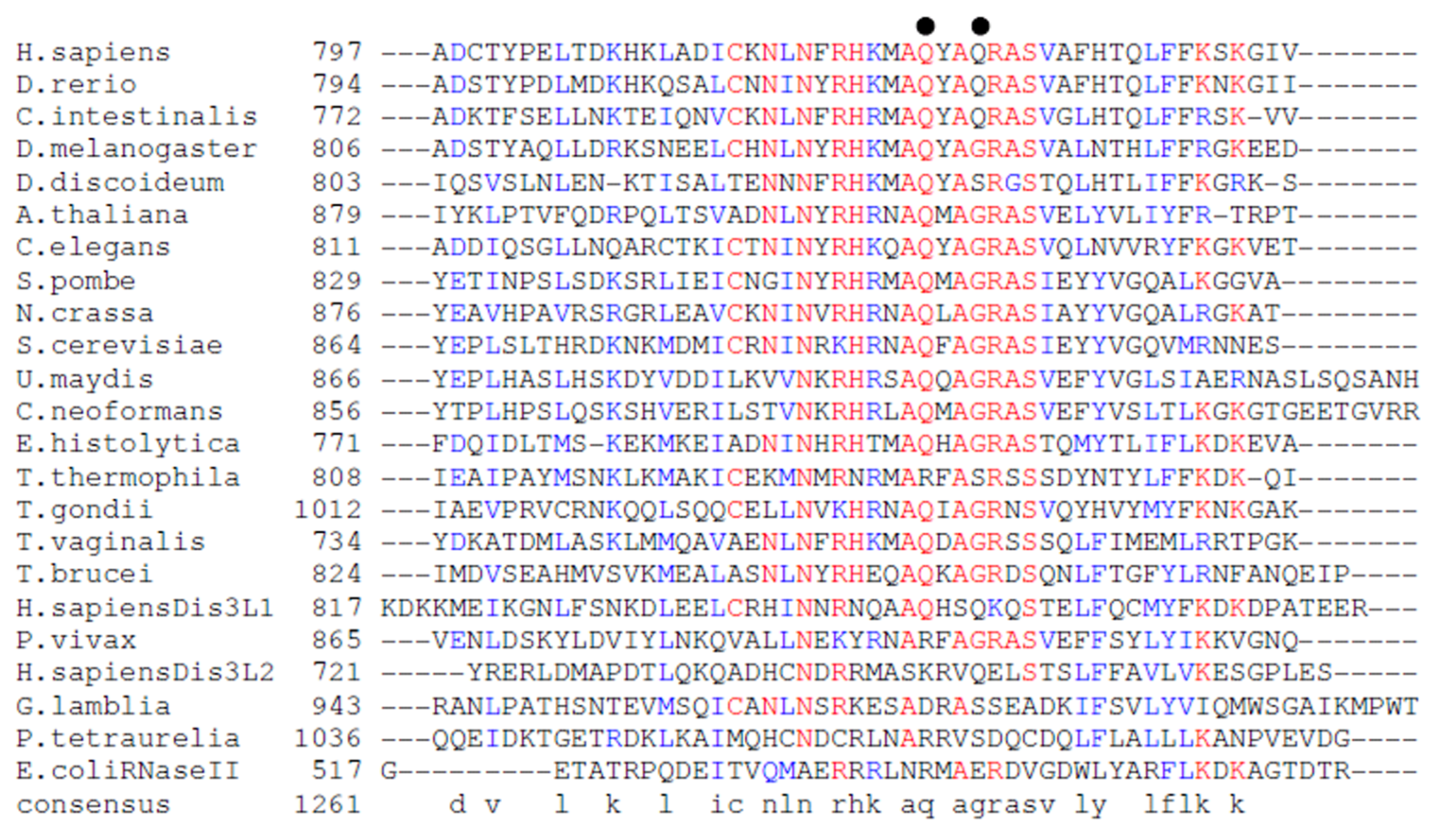

Supplement: Figure S1 — Partial multiple sequence alignment of Rrp44 homologues. The full length sequences of Rrp44 homologues were aligned using ClustalW (http://www.ebi.ac.uk/Tools/msa/clustalw2/) and boxshade (http://www.ch.embnet.org/software/BOX_form.html) with default settings. This figure shows only a small part of the alignment, in yeast Rrp44 Q892-G895 region. Positions of yeast Q892 and conserved G895 are indicated. Red: residues identical in at least half of the sequences. Blue: residues similar in at least half of the sequences. (TIF) [file pone.0076504.s001.tif]

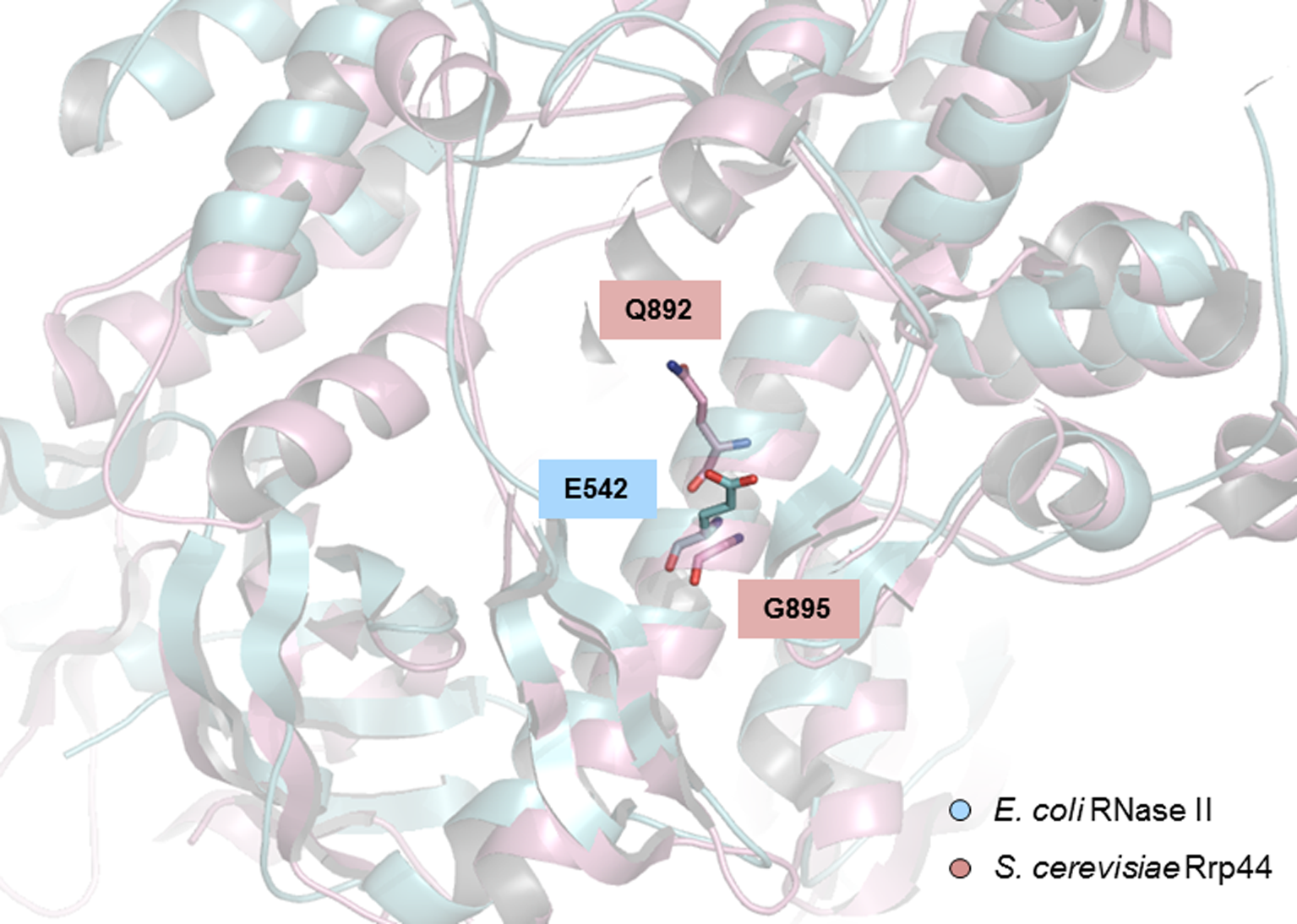

Supplement: Figure S2 — Partial multiple structural alignment of Rrp44 homologues. Superposition of E. coli RNase II (PDB ID 2IX1) [7] and S. cerevisiae Rrp44 (PDB ID 2VNU) [20] indicates that residue E.coli E542 and S. cerevisiae G895 residues occupy the same position in the space. Structures were rendered using PyMOL. (TIF) [file pone.0076504.s002.tif]

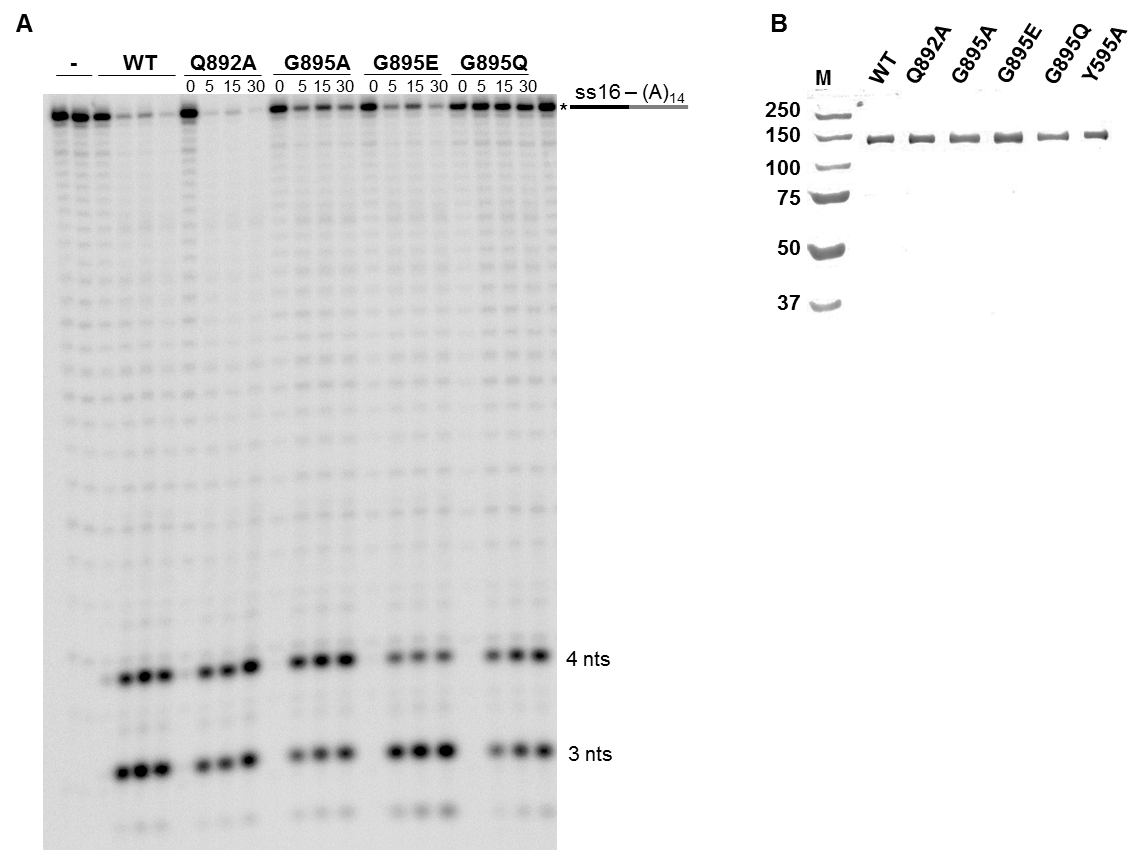

Supplement: Figure S3 — Exoribonuclease activity of Rrp44 and the different mutants. (A) Activity assays were performed as described in Materials and Methods using 20 nM of enzyme and 30-mer 5′-radioactively labelled RNA substrate (5′-CCCGACACCAACCACUAAAAAAAAAAAAAA-3′). Reactions were stopped and samples were taken at the time-points indicated. Length of substrates and degradation products are indicated in the figure. (B) SDS-PAGE analysis of wild-type and Rrp44 mutants. Equal amounts of mutant proteins were loaded into each lane; the gel was stained with Coomassie blue for detection. (TIF) [file pone.0076504.s003.tif]

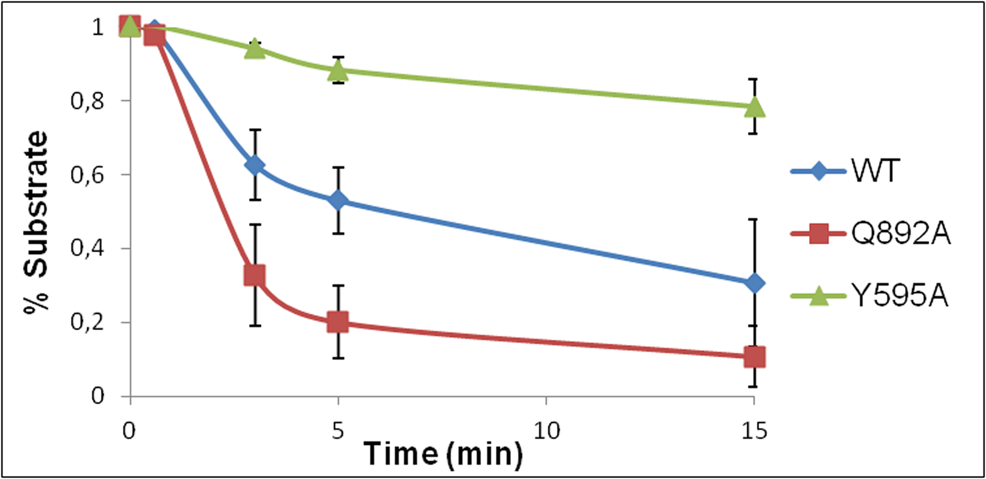

Supplement: Figure S4 — Exoribonuclease activity of wild-type Rrp44, Q892A and Y595A mutant. Exoribonucleolytic activity of the enzymes was determined by quantifying the fraction of full-length RNA lost using 20 nM of enzyme (WT, Q892A and Y595A) and 5′-radioactively labeled 30-mer oligoribonucleotide supplemented with 40 nM of non-radioactively (5′-CCCGACACCAACCACUAAAAAAAAAAAAAA-3′). Graphs depict the degradation of substrate at different time points calculated using Image Quant. Each value represents the average of three independent assays. (TIF) [file pone.0076504.s004.tif]

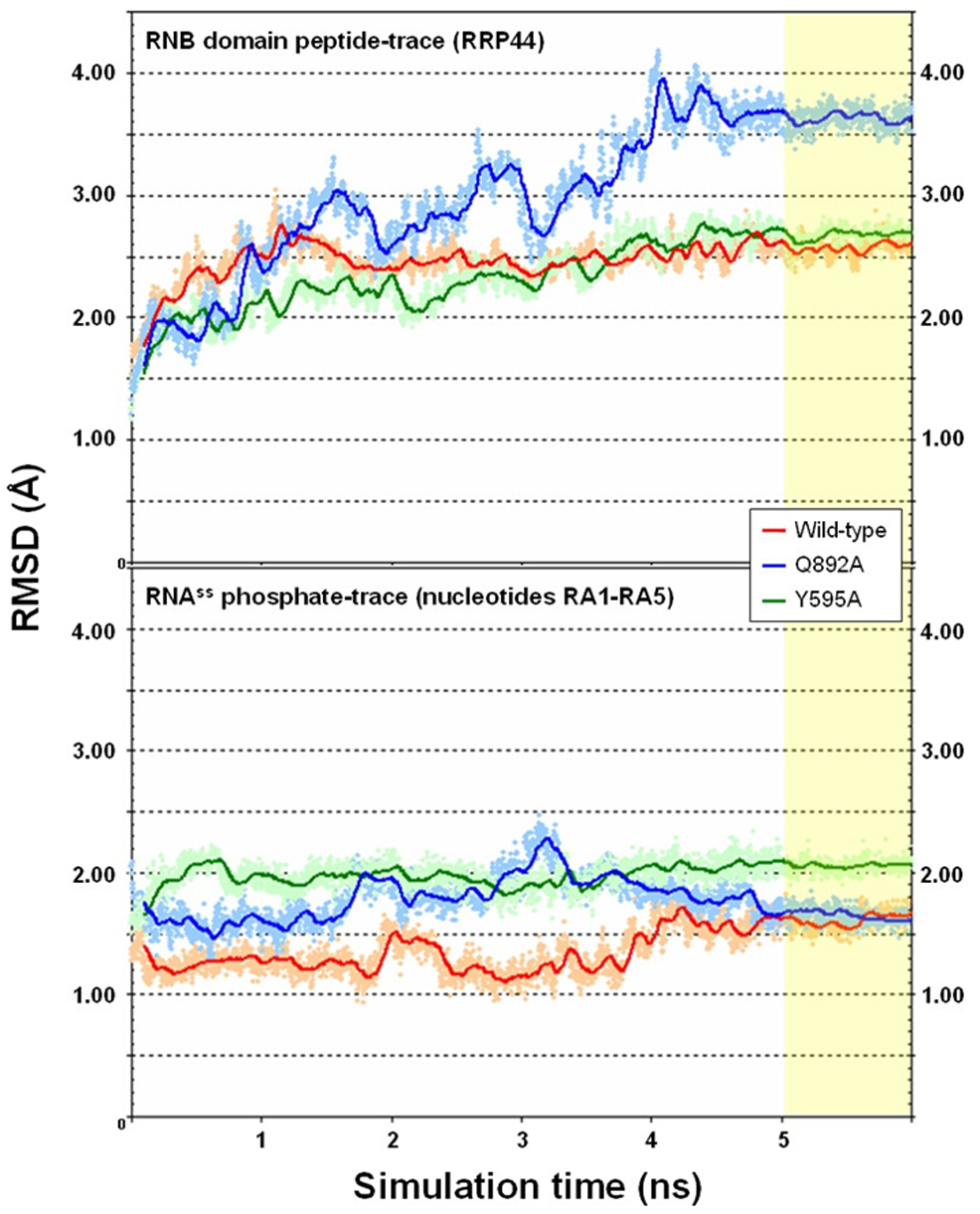

Supplement: Figure S5 — General structural deviations of RNB-ssRNA theoretical models from yeast Rrp44 wild-type and mutants. RMSD temporal profiles of RNB domain peptide-backbones (upper panel) and five most 3′ ssRNA nucleotides (RA1–RA5) phosphate-backbone (lower panel) traces. RMSD point values and running averages (over 20) plotted in colours red (wild-type), blue (Q892A) and green (Y595A). RMSD measurements to initial conformations, revealed some interesting differential responses during the last segment of the simulations (Table S1), highlighted in soft yellow colour. Data from Rrp44 Q892A suggest that this mutation might be able to induce significant variability in the peptide-backbone of the RNB domain, in comparison to the wild-type and Y595A mutant. On the other hand, little variations seem to be affecting the phosphate-backbone structure of ssRNA within the catalytic cleft (nucleotides RA1–RA5) in the three phenotypes, although subtle differences could be altering it when bound to Y595A mutant. (TIF) [file pone.0076504.s005.tif]
