# Supplementary material for: Modulating the RNA Processing and Decay by the Exosome: Altering Rrp44/Dis3 Activity and End-Product
Source: PLoS One. 2013 Nov 12;8(11):e76504. doi: 10.1371/journal.pone.0076504 (PMC3827031; doi:10.1371/journal.pone.0076504)
Supplement: Table S1 — Average RMSD values. Average RMSD values obtained from the last nanosecond of free molecular dynamics trajectories of the three yeast Rrp44-ssRNA interaction models calculated. Data correspond to peptide-backbone trace of RNB domains and nucleotides RA1–RA5 in RNA bound molecules respectively. (DOCX) [file pone.0076504.s006.docx]

|  | **Rrp44** | poly(A)-RNA |
| --- | --- | --- |
|  | RNB domain (Å) | RA1-RA5 (Å) |
| Wild-type | 2.58±0.08 | 1.59±0.07 |
| Y595A | 2.68±0.08 | 1.69±0.08 |
| Q892A | 3.63±0.10 | 2.07±0.06 |
